# Enhanced anti-tumor efficacy and mechanisms associated with docetaxel-piperine combination- *in vitro* and *in vivo* investigation using a taxane-resistant prostate cancer model

**DOI:** 10.18632/oncotarget.23235

**Published:** 2017-12-14

**Authors:** Chenrui Li, Zhijun Wang, Qian Wang, Rebecca Lucinda Ka Yan Ho, Ying Huang, Moses S.S. Chow, Christopher Wai Kei Lam, Zhong Zuo

**Affiliations:** ^1^ School of Pharmacy, Faculty of Medicine, The Chinese University of Hong Kong, Shatin, New Territories, Hong Kong SAR; ^2^ Key Laboratory for Space Bioscience and Biotechnology, School of Life Sciences, Northwestern Polytechnical University, Xi’an, Shaanxi, China; ^3^ Center for Advanced Drug Research and Evaluation, College of Pharmacy, Western University of Health Sciences, Pomona, CA, USA; ^4^ Department of Pharmaceutical Sciences, College of Pharmacy, Marshall B. Ketchum University, Fullerton, CA, USA; ^5^ State Key Laboratory of Quality Research in Chinese Medicines, Macau Institute for Applied Research in Medicine and Health, Macau University of Science and Technology, Taipa, Macau

**Keywords:** herb-drug interaction, piperine, docetaxel, taxane-resistant prostate cancer

## Abstract

Docetaxel (DTX) is widely used for metastatic castrated resistant prostate cancer, but its efficacy is often compromised by drug resistance associated with low intracellular concentrations. Piperine (PIP) could enhance the bioavailability of other drugs via the inhibition of CYPs and P-gp activities. Thus, we hypothesize a positive effect with the DTX-PIP combination on the anti-tumor efficacy and intra-tumor DTX concentrations in taxane-resistant prostate cancer. ICR-NOD/SCID mice implanted with taxane-resistant human prostate cancer cells were administrated with saline as well as PIP and DTX separately or in combination. The tumor growth was monitored together with intra-tumor concentrations of DTX. The inhibitory effects on CYPs and P-gp were further assessed in mouse liver microsome and MDCK-MDR1 cells. Compared with DTX alone, DTX-PIP combination significantly inhibited the tumor growth (114% vs. 217%, *p* = 0.002) with corresponding significantly higher intra-tumor DTX concentrations (5.854 ± 5.510 ng/ml vs. 1.312 ± 0.754 ng/mg, *p* = 0.037). The percentage of DTX metabolism was significantly decreased from 28.94 ± 1.06% to 18.14 ± 2.22% in mouse liver microsome after administration of PIP for two weeks. DTX accumulation in MDCK-MDR1 cell was significantly enhanced in the presence of PIP. Further microarray analysis revealed that PIP inhibited P-gp as well as CYP1B1 gene expression and induced a significant gene expression change relating to inflammatory response, angiogenesis, cell proliferation, or cell migration. In conclusion, DTX-PIP combination significantly induces activity against taxane-resistant prostate tumor. Such effect appeared to be attributed to the inhibitory effect of PIP on CYPs and P-gp activity as well as gene expression changes relating to tumorigenesis and cellular responses.

## INTRODUCTION

Prostate cancer is the most common non-cutaneous carcinoma in men. According to the latest data provided by American Cancer Society, prostate cancer has the most prevalent diagnoses (21%) with 26120 estimated deaths in 2016 in the US [1]. In China, prostate cancer also has high incidence and mortality [2]. Advanced metastatic prostate cancer, also called metastatic castrate-resistant prostate cancer (mCRPC), requires anticancer chemotherapy. Approved by US Food and Drug Administration in 2004, docetaxel (DTX) has been the mainline chemotherapy drug for treating mCRPC since it has the lowest cost and best cost/outcome as well as the highest relative value based on a simplified drug model [3]. However, the efficacy of DTX therapy is only about 50% initially which will inevitably develop chemo-resistance later on [4–7]. Due to these limitations, strategies to enhance the DTX response using compounds which have been already exposed to human subjects appears to be worthwhile as the drug development cost and time will be significantly reduced compared to new medicinal entity.

A common mechanism that contributes to the development of resistance is a reduction of the intracellular drug concentration, which might be due to the low uptake of drug, extensive metabolism of drug, or drug efflux out of the cancer cells [5, 8, 9]. DTX undergoes extensive hepatic metabolism mediated by cytochromes P450 CYPs, especially the CYP 3A subfamily [10, 11]. There are four metabolites identified in human plasma and feces which have much lower activity than that of DTX [12, 13]. Reduction in the intra-cellular concentration is well known to occur with the high expression of efflux transporters such as P-gp or MDR1 encoded by the *ABCB1* gene, multidrug resistance proteins (MRPs, encoded by *ABCC* gene), as well as breast cancer resistance protein (BCRP, encoded by *ABCG2* gene) [5]. Thus, strategies to inhibit both the metabolism and efflux transporters could enhance drug concentration and overcome drug resistance. The above concept has been substantiated from a number of published reports on DTX resistance. Ketoconazole, a potent CYP3A4 inhibitor, administrated at 200 mg once daily for three days has been reported to significantly reduce the clearance of DTX by 49% in cancer patients (*p* = 0.018) [14]. On the other hand, St John’s wort, a CYP 3A4 inducer, has been reported to significantly reduce the AUC_0→∞_ of DTX in cancer patients and its combination use with DTX should be avoided [15]. Moreover, a synthesized naphthoflavone as CYP1B1 inhibitor could decrease the IC_50_ of DTX in MCF7/1B1 cells [16]. Although Tripterygium wilfordii is attractive in combination DTX for potential mCRPC resistant to DTX, Tripterygium wilfordii as an extract has problem of quality control and therapeutic monitoring [17]. Therefore, a single natural compound which can overcome DTX resistance will be more attractive.

Piperine (1-piperoyl piperidine, PIP), an abundant alkaloid compound in *Piper longum* L. (long pepper) and *Piper nigrum* L. (white or black pepper), exhibits a number of beneficial effects including anti-cancer, anti-inflammation, immunomodulatory, antispasmodic and anti-secretory effects [18–22]. In addition to these pharmacological effects, PIP has been found to significantly inhibit the activities of metabolic enzymes such as CYPs, UDP-glucuronosyltransferase, sulfotransferase, aryl hydroxyoase and O-deethylase [23–25], and selectively inhibit the function of CYP3A4 [26]. Besides, the backbone of PIP has been adopted in designing and developing non-toxic P-gp inhibitors [27]. PIP also has been reported to significantly inhibit the transport of digoxin and cyclosporine A in a Caco-2 monolayer model with IC_50_ of 15.5 and 74.1 μM, respectively [28]. PIP at the dose of 112 mg/kg/day for 14 days has led to a decreased expression of hepatic P-gp but increased level of intestinal P-gp [29]. In fact, a number of studies have reported PIP as a bioavailability enhancer for a number of drugs including rosuvastatin, puerarin, diltiazem, domperidone, linarin and etoposide via its inhibition on CYP activity and/or P-gp function [30–35]. Importantly, treatment of PIP at 50 μM has been reported to significantly enhance the sensitivity to doxorubicin in anti-cancer drug resistant cell lines by 32 folds (MCF-7/DOX) and 14 folds (A-549/DDP), respectively [36]. In addition, it was reported that co-administration of PIP could increase the systemic exposure of DTX and improve the anti-cancer effect in C.B17/Icr-scid mice with tumor xenograft of PC3 cells [37]. In our previous study, PIP has been found to be able to reverse the chemoresistance of PC3-TxR cells (DTX resistant prostate cancer cells) to DTX (Supplementary Figure 1). However, whether co-administration of PIP with DTX can overcome DTX resistant tumor remains unknown. Thus, the main purpose of the present study is to investigate the effect of DTX-PIP combination in taxane-resistant tumor using a mice xenograft model implanted with PC3-TxR cells.

The percentage decrease in tumor size was monitored after the administration of DTX in the presence or absence of PIP and the concentration of DTX in tumor or other tissues were quantified by LC/MS/MS. To mechanistically further investigate the effects of PIP on DTX metabolism and intracellular exposure, enzymatic incubation study with mice liver microsome and cell uptake study in MDCK cells transfected with human MDR1 gene were adopted. In addition, the molecular biological mechanisms were primarily determined using the microarray technology.

## RESULTS

### *In vivo* verification of taxane-resistant prostate mice model

**Figure 1:**
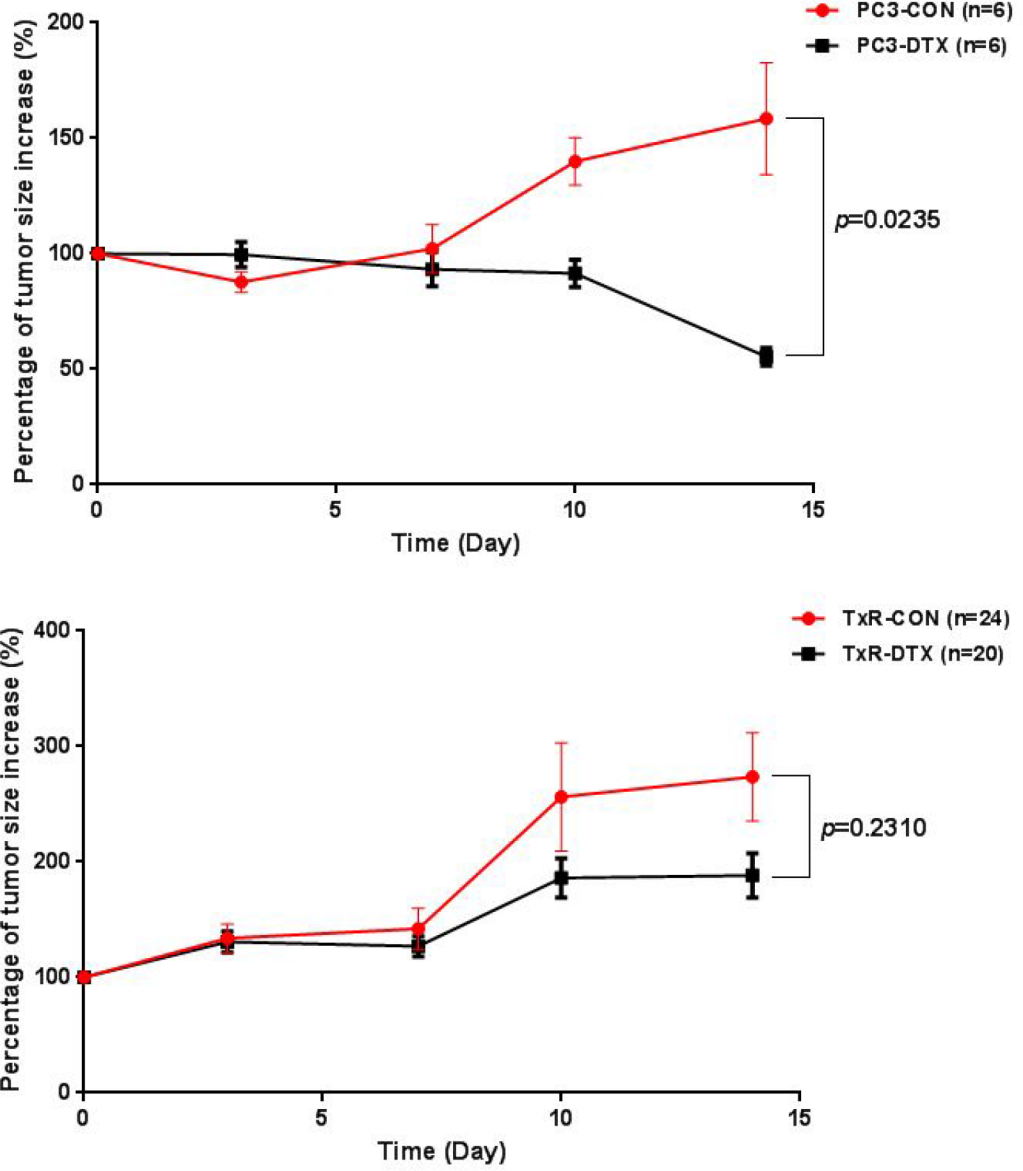
Plot of percentage of tumor size increase (%) versus time in mice with PC3 tumor xenografts (upper) and TxR tumor xenografts (lower)

The resistance of PC3-TxR cell line to the treatment of DTX was investigated in 6 to 8-week-old male ICR-NOD/SCID mice implanted with PC3 and PC3-TxR tumor xenografts, respectively (*n* = 3 in each group). Two weeks after xenograft implantation when the tumor size in the mice reached about 100 mm^3^, treatment was initiated with vehicle solvent or DTX to the PC3 and PC3-TxR implanted groups. The percentage of tumor size increase was calculated and plotted versus time. As shown in Figure 1, tumor sizes increased with time in all groups except for the PC3-DTX group in which DTX significantly inhibited the tumor size increase in PC3 tumor xenografts (55% for PC3-DTX *vs.* 158% for PC3-CON, *p* = 0.024), whereas treatment with DTX showed no inhibitory effect on the tumor growth in TxR tumor xenografts. These results indicated that PC3 cells were sensitive to the treatment of DTX, whereas mice implanted with PC3-TxR tumor xenografts showed significant resistance to the treatment of DTX.

### *In vivo* evaluation of PIP on the anti-tumor effect of DTX and body weight in TxR implanted tumor

**Figure 2:**
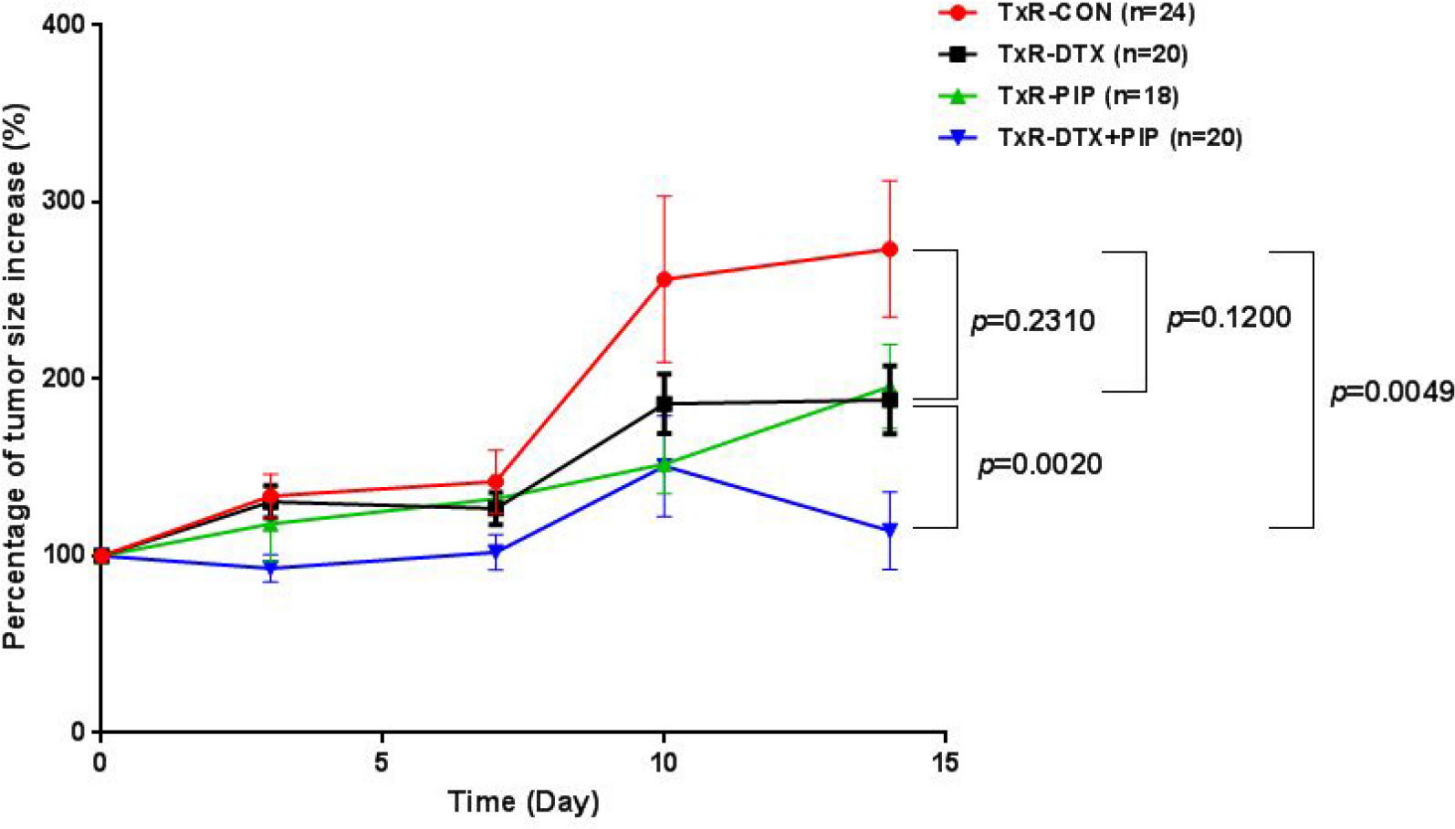
Plot of percentage of tumor size increase (%) versus time in mice with TxR tumor xenografts after the treatment with saline (*n* = 24), DTX (20 mg/kg, *n* = 20), PIP (50 mg/kg, *n* = 18), or co-administration of DTX and PIP (20 mg/kg DTX and 50 mg/kg PIP, *n* = 20) Abbreviation: DTX = docetaxel; PIP = piperine.

As shown in Figure 2, DTX treatment could not inhibit the tumor growth in TxR tumor bearing mice. Since PIP is an inhibitor of P-gp and CYP3A4 and it has been reported to enhance the bioavailability of several drugs, the effect of DTX-PIP combination was examined in mice with TxR tumor xenografts. Two weeks after drug administration, neither DTX (217% vs 274%, *p* = 0.231) nor PIP alone (196% vs. 274%, *p* = 0.120) could inhibit tumor size increase compared with that from the vehicle control group. However, co-administration of DTX and PIP resulted in very significant inhibitory effect on tumor growth compared with vehicle control (114% *vs.* 274%, *p* = 0.005) or compared with DTX alone (114% *vs.* 217%, *p* = 0.002), suggesting that DTX-PIP combination could enhance the anti-tumor efficacy against chemo-resistant prostate tumor. Furthermore, the bodyweights of all four groups showed a tendency of decrease with time (Supplementary Figure 2). Compared with the control group, the percentage of bodyweights in mice treated with DTX (79% *vs.* 96%, *p* = 0.0002) or the combined administration of DTX and PIP (85% *vs.* 96%, *p* = 0.008) were all significantly decreased, whereas the bodyweight of mice treated with PIP alone were comparable to those of control group (98% *vs.* 96%, *p* = 0.590). Moreover, Box and Whiskers plots with 5–95 percentile were constructed to compare Area under the Curve (AUC) of percent tumor growth in four treated groups (Figure not shown). AUC values were calculated using a linear trapezoid method. The AUC of the group treated with DTX-PIP combination was significantly lower than those treated with DTX or PIP alone (*p* = 0.002, 1-way ANOVA followed by Bonferroni multi-comparison). No statistical significance was observed between the groups treated by DTX or PIP alone in comparison with the vehicle control (*p* > 0.05, 1-way ANOVA).

### Effect of PIP on the DTX tissue distribution in TxR tumor bearing mice

On Day 14, mice were sacrificed two hours after the intravenous bolus administration of DTX. Their plasma samples and tissue homogenates were treated by a protein precipitation method and the concentration of DTX therein was quantified using LC/MS/MS. The concentrations of DTX in various tissues were calculated and listed in Table 1. It was found that DTX-PIP combination significantly increased the concentrations of DTX in tumor (5.854 ng/mg *vs.* 1.312 ng/mg, *p* = 0.037), liver (2.782 ng/mg *vs.*1.080 ng/mg, *p* = 0.006) and plasma (1448.432 ng/ml *vs.*703.847 ng/ml, *p* = 0.006) compared to those treated with DTX alone. The concentrations of DTX in the brain (0.051 ng/mg *vs.*0.022 ng/mg, *p* = 0.108) and kidneys (5.980 ng/mg *vs.*2.322 ng/mg, *p* = 0.081) showed a trend increase after DTX-PIP combination. These results indicate that PIP could increase the *in vivo* exposure of DTX and the elevated concentration of DTX in tumor tissue might contribute to its enhanced anti-tumor effect after combined use of DTX and PIP.

**Table 1:** Comparison of DTX concentration sin plasma and various tissues between TxR mice treated with DTX and combination of DTX and PIP

Groups	Tissue concentration (ng/mg)					Plasma concentration (ng/ml)
	Tumor	Brain	Kidney	Lung	Liver	
**TxR-DTX (*n* = 10)**	1.312 ± 0.754	0.022 ± 0.009	2.322 ± 1.447	5.158 ± 2.886	1.080 ± 0.763	703.847 ± 311.129
**TxR-DTX+PIP (*n* = 10)**	5.854 ± 5.510^*^	0.051 ± 0.047	5.980 ± 5.234	4.991 ± 3.133	2.782 ± 0.891^**^	1448.432 ± 698.493^**^

Note: Comparisons were conducted by Student’s *T*-test. ^*^p < 0.05; ^**^p < 0.01; ^***^p < 0.001.

Abbreviation: DTX = docetaxel; PIP = piperine.

### P-gp inhibition and DTX accumulation in MDCK/MDR1 cells and PC3-TxR cells

The induction of efflux membrane transporters, such as P-gp, MRPs and BCRP, contribute to the low drug concentration, which could result in chemotherapy resistance. Since PIP is a well-known P-gp inhibitor, its effects on the intracellular concentration of DTX in MDCK cells transfected with human MDR1 gene as well as PC3-TxR cells were investigated.

### Enhanced uptake of DTX by PIP in MDCK/MDR1 cells

**Figure 3:**
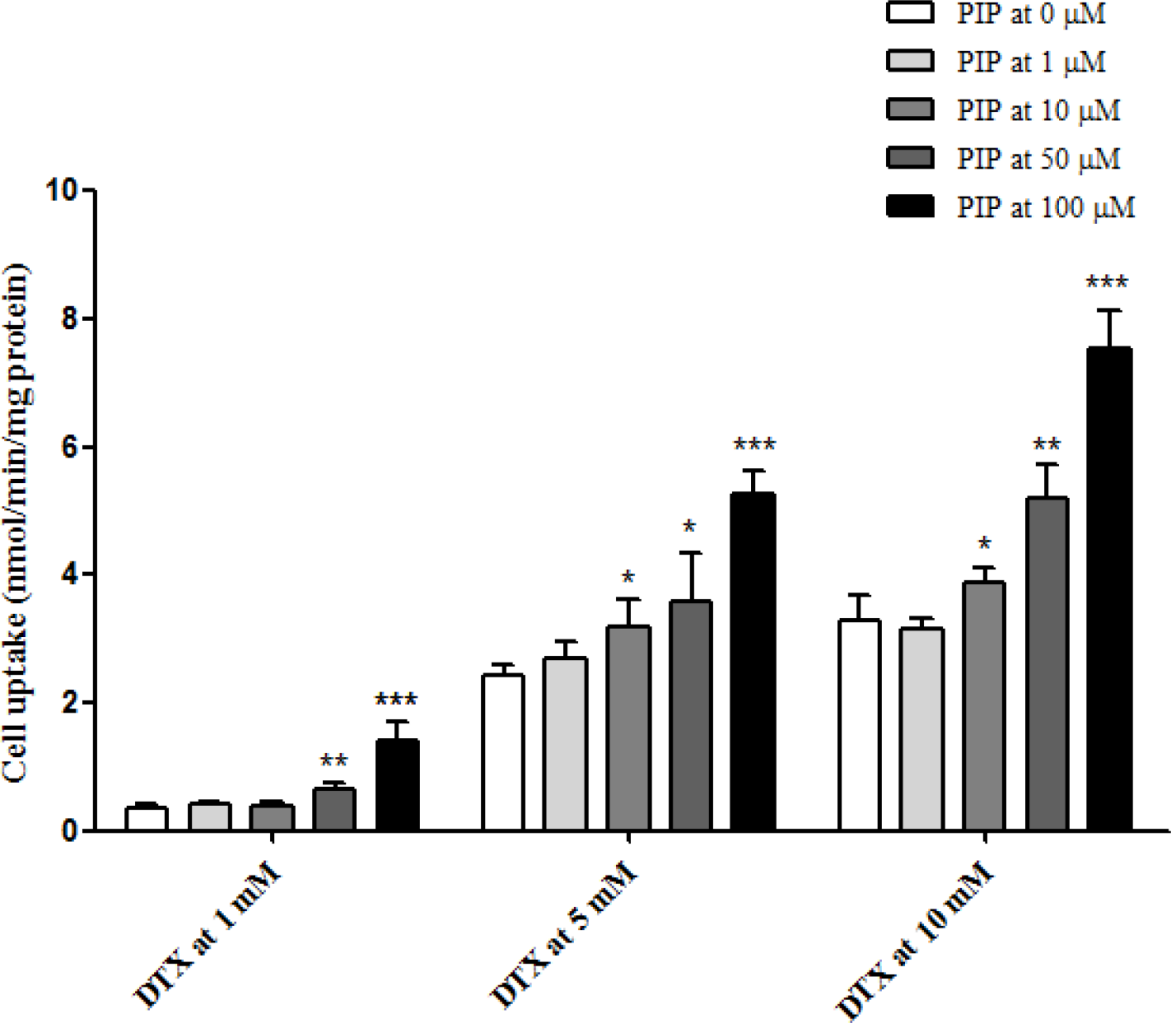
Uptake of DTX at 1, 5 and 10 μM in MDCK/MDR1 cells after pre-incubation with PIP at 1, 10, 50 and 100 μM (n = 4 each)

As shown in Figure 3, cell uptake of DTX at 1, 5 and 10 μM in MDCK cells was calculated and plotted with and without pre-incubation of PIP at various concentrations. In the absence of PIP, the cell uptake of DTX was increased with loading concentrations (0.35 ± 0.05 nmol/min/mg protein at 1 μM, 2.44 ± 0.14 nmol/min/mg protein at 5 μM and 3.30 ± 0.38 nmol/min/mg protein at 10 μM). Furthermore, it was found that PIP could significantly increase the uptake of DTX and this effect was enhanced when the concentration ratio increased. Loaded at 1 μM, the intracellular concentration of DTX was elevated by 84% with 50 μM of PIP and 305% with 100 μM of PIP. After pre-incubation with PIP at 50 mM, the cell uptake of DTX increased from 2.44 ± 0.14 to 3.57 ± 0.75 nmol/min/mg protein at the loading concentration of 5 μM and from 3.30 ± 0.38 to 5.20 ± 0.53 nmol/min/mg protein at 10 μM. PIP at 100 μM could significantly increase the uptake of DTX by 116% at 5 μM and by 129% at 100 mM. These results suggested that the intracellular concentration of DTX was significantly enhanced by PIP via the inhibition of P-gp activity.

### Intracellular DTX accumulation in PC3-TxR cells

**Figure 4:**
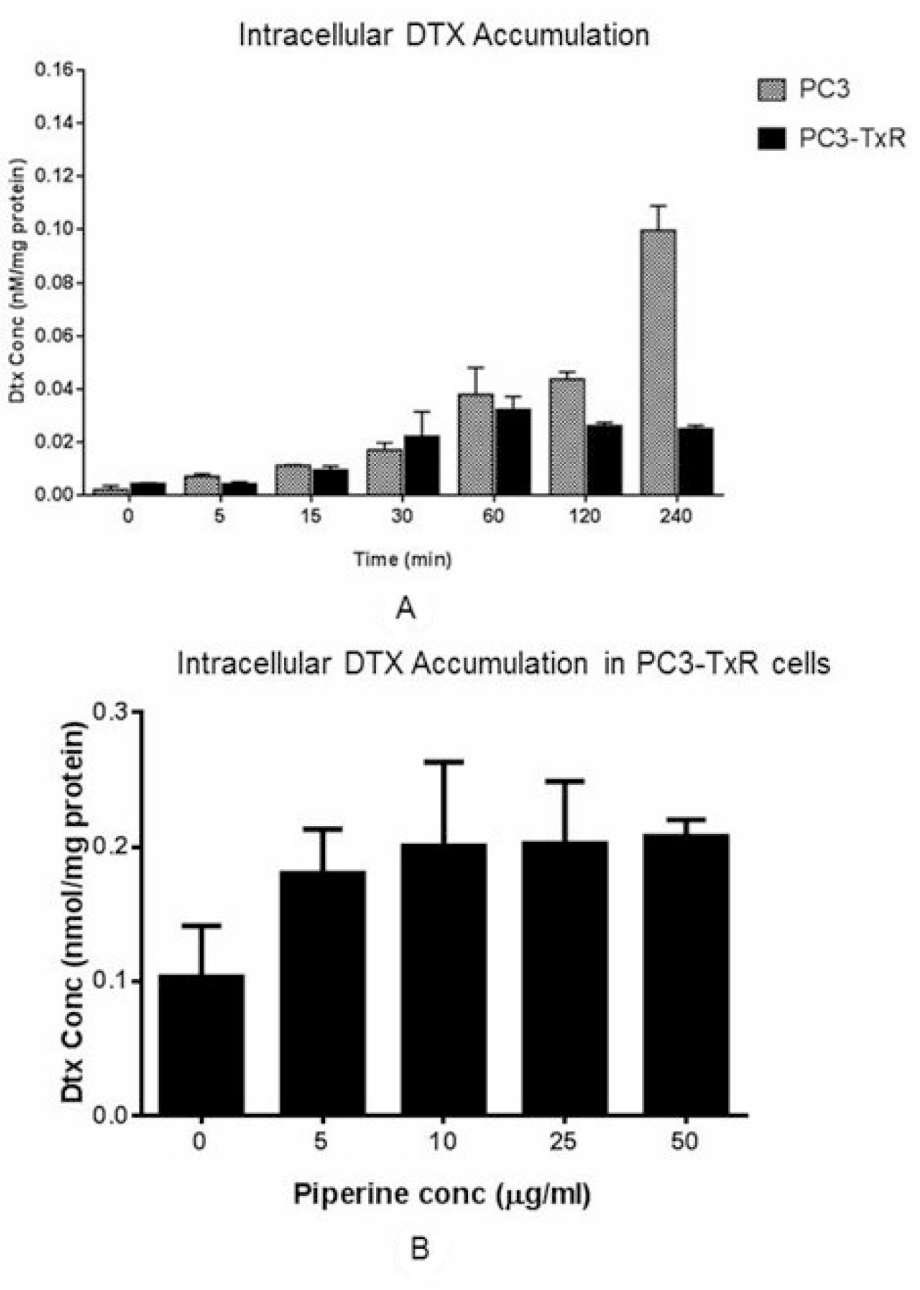
DTX accumulation **(A)** at 100 nM concentration in PC3-TxR and PC3 cells at different time intervals, and **(B)** in PC3-TxR cells in absence and presence of various concentrations of PIP (*n* = 4). Abbreviation: DTX = docetaxel; PIP = piperine**.**

We found that the intracellular DTX concentration increased along with time in PC3 cells, while such concentration was stable after 2 hours in PC3-TxR cells. DTX concentration in PC3 cells was significantly higher than that in PC3-TxR cells indicating a significant efflux of DTX (Figure 4A). Since DTX is a substrate of P-gp which is overexpressed in PC3-TxR cells, lower concentration of intracellular DTX concentration was a result of P-gp induction at the baseline. However, the intracellular DTX concentration increased when combined with PIP in the PC3-TxR cells. At 50 μg/ml, the intracellular DTX concentration was increased to a level similar to that in the positive control (10 μg/ml of PSC833, a P-gp inhibitor; Figure 4B).

### Inhibition on the liver metabolism of DTX by PIP

**Figure 5:**
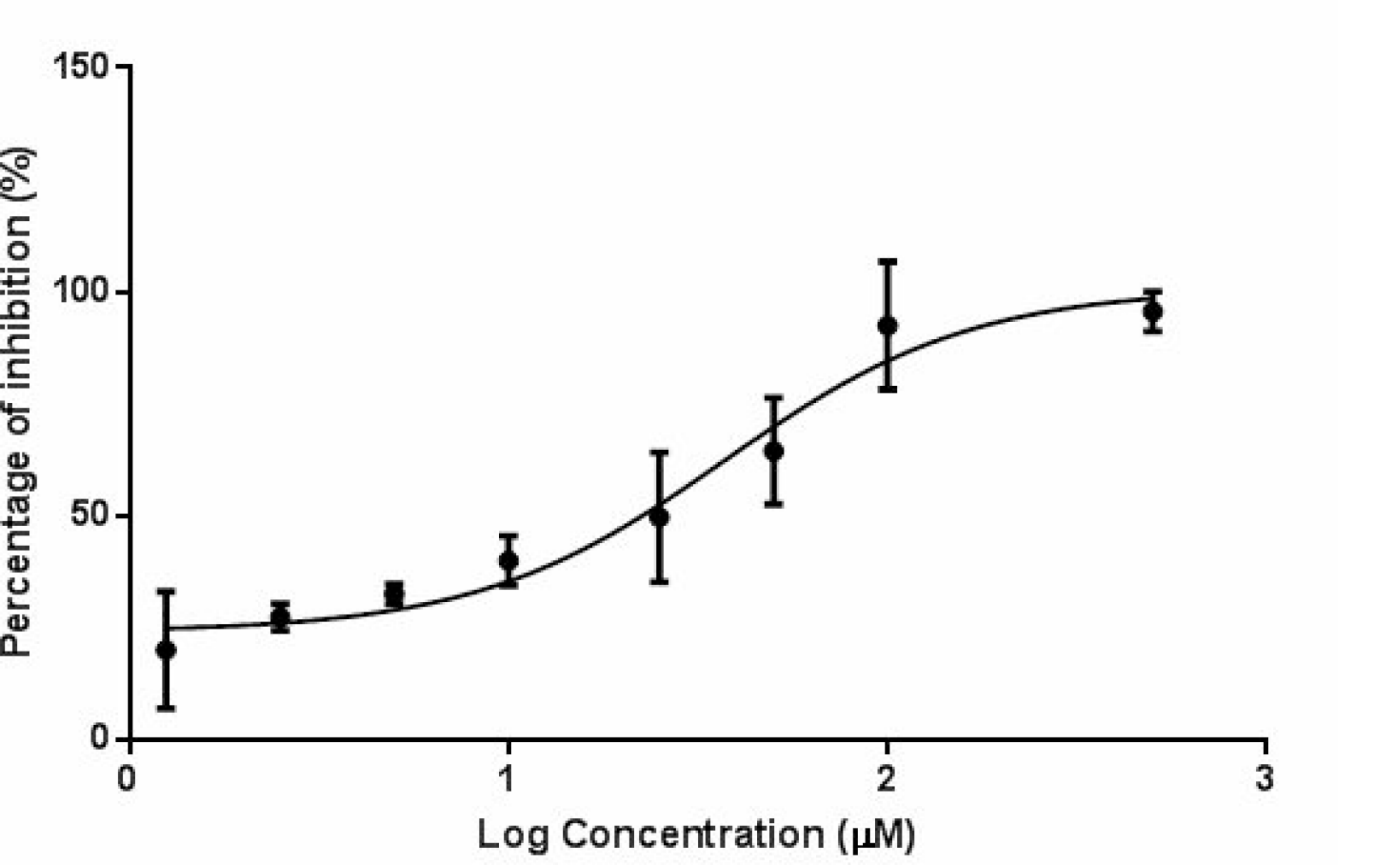
Percentage of inhibition on the hepatic metabolism of DTX (2 μM) by PIP at various concentrations (*n* = 3) Abbreviation: DTX = docetaxel; PIP = piperine.

DTX undergoes extensive Phase I metabolism that is mediated mainly by the cytochrome P450 3A subfamily that is abundant in liver microsomes. Mouse liver microsomes were prepared and the inhibitory effect of PIP on the hepatic metabolism of DTX was determined *ex vivo* and *in vitro*. It was found that two weeks of PIP administration to ICR-NOD/SCID mice significantly decreased the *ex vivo* percentage of metabolism of DTX compared with the vehicle control (18.14 ± 2.22% *vs.* 28.94 ± 1.06%, *p* = 0.011). In the liver microsomes, PIP also inhibited the metabolism of DTX in the loading concentration ranging from 1.25 μM to 200 μM. The plot of percentage of inhibition versus log concentration of PIP is shown in Figure 5 and the IC_50_ was calculated to be 37.28 μM.

### Study of molecular mechanisms

In addition to the inhibition of P-gp, other potential molecular biological targets were identified using Microarray study.

### Quality assessment

The average intensities of the array were similar and no array was suspected to be an outlier (Supplementary Figure 3). Visual inspection of the principal component analysis (PCA) plot yielded a straightforward diagnosis of the sources of variance in these datasets. The PCA is based on log2 intensity as shown in Supplementary Figure 4. Samples for the same treatment group were clustered together indicating good reproducibility of microarray experiments. Hierarchical clustering analysis was also used to assess the overall quality of the microarray data. The three replicates in each treatment group were clustered together in terms of log2 gene expression. The expression profiles in group treated with PIP (high concentration) were dramatically different from that of the control group.

### DEGs determination

Greater number of differentially expressed genes was identified in the PIP high concentration group than the low-dose group. The number of DEGs varied dramatically depending on the treatments. The numbers of DEGs showed a clear dose dependent manner, with an increase from low dose to high dose (69 and 363 up-regulated probes for low and high dose group respectively, see Table 2). Using a filter criterion of at least a 2-fold change with *p* < 0.05, the number of genes with changes in expression in PC3-TxR treated with PIP compared to the control group was determined. A total of 363 and 238 probes were up-regulated and down regulated respectively in high dose PIP group, while 69 and 45 probes up-regulated and down regulated respectively in low dose PIP group (Table 2).

**Table 2:** Differentially expressed genes/probes in two different dosage groups

Comparison	Up-regulated	Down-regulated
PPR_H vs. Control	363 probes	238 probes
PPR_L vs. Control	69 probes	45 probes

### Pathway analysis

A gene ontology-based (GO) analysis of biological properties was used to determine the functional annotation of the treatment of PIP. The differentiated expressed genes were categorized into the functional groups with the top 10 of each group listed in Table 3 and Supplementary Tables 1 and 2. Most of these genes were associated with the inflammatory response, angiogenesis, cell proliferation, or cell migration.

**Table 3:** Gene ontology results (PPR_H: high dose piperine group, 100 μg/ml; PPR_L: low dose piperine group, 50 μg/ml)

Category	Term	*P* Value	Category	Term	*P* Value
PPR_H vs. Control (up regulation)			PPR_H vs. Control (down regulation)		
GOTERM_BP_FAT	GO:0006357∼regulation of transcription from RNA polymerase II promoter	1.06E-06	GOTERM_MF_FAT	GO:0003723∼RNA binding	3.83E-10
GOTERM_MF_FAT	GO:0016564∼transcription repressor activity	1.49E-06	GOTERM_MF_FAT	GO:0000166∼nucleotide binding	3.45E-06
GOTERM_BP_FAT	GO:0043067∼regulation of programmed cell death	2.81E-06	GOTERM_BP_FAT	GO:0006986∼response to unfolded protein	7.13E-05
GOTERM_BP_FAT	GO:0010941∼regulation of cell death	3.02E-06	GOTERM_BP_FAT	GO:0016071∼mRNA metabolic process	7.74E-05
GOTERM_BP_FAT	GO:0031328∼positive regulation of cellular biosynthetic process	3.81E-06	GOTERM_BP_FAT	GO:0051789∼response to protein stimulus	9.16E-05
GOTERM_BP_FAT	GO:0009891∼positive regulation of biosynthetic process	4.91E-06	GOTERM_BP_FAT	GO:0006396∼RNA processing	1.01E-04
GOTERM_BP_FAT	GO:0010557∼positive regulation of macromolecule biosynthetic process	5.53E-06	GOTERM_BP_FAT	GO:0007049∼cell cycle	2.27E-04
GOTERM_BP_FAT	GO:0042981∼regulation of apoptosis	7.06E-06	GOTERM_BP_FAT	GO:0010033∼response to organic substance	2.66E-04
GOTERM_BP_FAT	GO:0006355∼regulation of transcription, DNA-dependent	9.12E-06	GOTERM_CC_FAT	GO:0043233∼organelle lumen	2.93E-04
GOTERM_BP_FAT	GO:0006357∼regulation of transcription from RNA polymerase II promoter	1.06E-06	GOTERM_BP_FAT	GO:0006397∼mRNA processing	3.47E-04

PPR_L vs. Control (up regulation)			PPR_L vs. Control (down regulation)		
GOTERM_MF_FAT	GO:0030528∼transcription regulator activity	0.0034	GOTERM_BP_FAT	GO:0042493∼response to drug	5.23E-04
GOTERM_MF_FAT	GO:0003700∼transcription factor activity	0.0039	GOTERM_BP_FAT	GO:0032570∼response to progesterone stimulus	5.62E-04
GOTERM_MF_FAT	GO:0043565∼sequence-specific DNA binding	0.0088	GOTERM_BP_FAT	GO:0042445∼hormone metabolic process	8.40E-04
GOTERM_BP_FAT	GO:0031667∼response to nutrient levels	0.0141	GOTERM_BP_FAT	GO:0050921∼positive regulation of chemotaxis	0.0012
GOTERM_BP_FAT	GO:0048514∼blood vessel morphogenesis	0.017	GOTERM_BP_FAT	GO:0010033∼response to organic substance	0.0013
GOTERM_BP_FAT	GO:0009991∼response to extracellular stimulus	0.0189	GOTERM_BP_FAT	GO:0050920∼regulation of chemotaxis	0.0014
GOTERM_BP_FAT	GO:0001568∼blood vessel development	0.0251	GOTERM_BP_FAT	GO:0006357∼regulation of transcription from RNA polymerase II promoter	0.0014
GOTERM_BP_FAT	GO:0045449∼regulation of transcription	0.0257	GOTERM_BP_FAT	GO:0001936∼regulation of endothelial cell proliferation	0.0014
GOTERM_BP_FAT	GO:0001944∼vasculature development	0.0267	GOTERM_BP_FAT	GO:0010038∼response to metal ion	0.0015
GOTERM_MF_FAT	GO:0016564∼transcription repressor activity	0.0299	GOTERM_BP_FAT	GO:0048520∼positive regulation of behavior	0.0016

### Dose dependent DEGs

Table 4 lists the dose-dependent DEGs. Five genes of each were significantly downregulated and upregulated in both low and high concentrations of PIP. Although CYP1B1 and TCDD-inducible poly (ADP-ribose) polymerase did not show obvious dose dependent manner, the changes of their expression were very significant. Therefore, these two genes were also included in Table 4. Among these genes, PIP can remarkably reduce the expression of CYP1B1 at both low and high dose treatment. The overexpression of CYP1B1 has been found to be associated with DTX resistance in both preclinical and clinical studies [38–40]. Thus, this can be another mechanism of chemosensitizing effect of PIP on PC3-TxR cells.

**Table 4:** List of dose dependent DEGs

	Low dose	High dose
CYP1B1	−5.1	−4.3
Keratin associated protein 2–3	−3.3	−4.7
TCDD-inducible poly(ADP-ribose) polymerase	−2.8	−2.6
Thrombospondin 1	−2.2	−2.4
Keratin associated protein 3–1	−2.0	−2.7
O-linked N-acetylglucosamine (GlcNAc) transferase	2.0	2.3
Fatty acid binding protein 4, adipocyte	2.5	1.9
Angiopoietin-like 4	2.3	1.6
Sodium channel, non-voltage-gated 1, gamma subunit	2.5	1.5
Metastasis associated lung adenocarcinoma transcript 1 (non-protein coding)	3.0	4.1

## DISCUSSION

Our present study confirmed a dual effect in enhancing DTX concentration when administrated as the DTX-PIP combination. PIP was found to inhibit DTX metabolism in the present study. Such finding is consistent with published report of PIP inhibition on CYP isoenzymes leading to enhanced exposure of ibuprofen, nateglinide and DTX [41–43]. In addition, we also found PIP inhibited the transporter activity (possibly P-gp activity) leading to increased intracellular concentration of DTX in a concentration dependent manner. Thus, we believe that the dual action of PIP by inhibiting DTX metabolism and P-gp activity could lead to enhanced DTX concentration, resulting in enhanced cytotoxicity and overcoming DTX resistance in prostate tumor, which was also suggested by others [44]. As to the tissue distribution of DTX, it was found that its concentration in lung was much higher than those in liver, brain, tumor and kidney. As a substrate of P-gp, the variation in the tissue distribution of DTX might be due to the expression level of P-gp in various organs. It was reported that P-gp is mainly expressed in small intestine, liver, kidney and brain in both human and mice [45, 46] and PC3-TxR cell line has high expression of P-gp. Therefore, the tissue distribution of DTX is expected to be lower in the P-gp expressed organ such as liver, kidney, brain and tumor than other organs with limited expression such as lung.

Other potential mechanisms of DTX-PIP combination were investigated based on microarray techniques. Pathway analysis showed that certain pathways relating to cell proliferation and cancer development were affected by the treatment of PIP indicating a potential activity in cancer therapy. Although the exact pathways involved in the chemoresistance were not identified, the microarray data indicated that PIP could suppress certain genes. For example, inhibition of CYP1B1 in tumors could offer a specific mechanism for overcoming the resistance to DTX and other drugs [47, 48]. The resistance could be attributed to the unspecific binding of DTX to CYP1B1 or hormone metabolism [49]. The inhibition of CYP1B1 could be one of the potential mechanisms as the suppression of upregulated CYP1B1 showed a chemosensitizing effect of DTX in PC3 resistance cell lines compared to sensitive cells.

The *in vivo* inhibitory efficacy of PIP has been extensively reported in several lung cancer animal models including C57BL/6 mice implanted with B16F-10 cancer cells and mice fed with benzo(a)pyrene [50–52]. In prostate cancer studies, PIP has been found to inhibit the proliferation of LNCaP, PC3, 22RV1 and DU-145 prostate cancer cells [53]. However, their *in vivo* effects are less consistent. The tumor volume as well as tumor mass was significantly reduced in nude mice implanted LNCaP (androgen dependent) and DU-145 (androgen independent) prostate cancer cells, but not in CB17/Icr-scid mice implanted with PC3 cells [53, 37]. In the current study, PIP alone (at the dose of 50 mg/kg for two weeks) has not been found to significantly reduce the percentage of tumor size increase in the TxR tumor xenograft (196 *vs.* 274%, *p* = 0.120). Such different *in vivo* prostate cancer study results might be due to diverse cell line responses as well as resistance. Thus, careful *in vivo* efficacy with mechanistic study and concentration determination of the relevant compounds are important. In addition, the dose of DTX was chosen based on literature reports [17, 54]. With regard to the dose of PIP, we firstly tried 100 mg/kg, the same dose as Makhov’s study in which PIP was used as a chemosensitizer to DTX [37]. Nevertheless, PIP at 100 mg/kg caused severe excitation responses in ICR-NOD/SCID mice implanted with taxane-resistant prostate cancer. Therefore, the dose of PIP was reduced to 50 mg/kg. Besides, Makhov *et al.* used PC3 cells (not chemoresistant) for cancer inoculation, whereas we used PC3-TxR cells (chemoresistant) which might be more aggressive and induce the incompliance of mice at a higher dose.

PIP is an alkaloid constituent abundant in *Piper longum* L. (long pepper) and *Piper nigrum* L. (white or black pepper) which are consumed as spicy seasoning for a long history especially in Asian countries [55, 56]. Besides, the acute toxicity studies of PIP revealed that its i.g. LD_50_ was 514 mg/kg in female Fischer rats, suggesting its safety after oral administration [57]. Although the anti-tumor effect of PIP was not significant in TxR implanted mice, PIP was effective for maintenance of body weight during our study. Besides, it was noticed that the mice conditions were much better in the PIP treated group with more activities and movements, which could be due to the beneficial effects of PIP on immune, cardiovascular system and central nervous system [20, 58, 59]. With human exposure history of PIP (in the form of pepper) and potential activity for DTX resistant mCRPC, it will be attractive to develop a DTX-PIP combination product in potential treatment of DTX resistant mCRPC.

## METHODS

### Materials and reagents

Piperine (PIP, purity >97%) and glibenclamide were obtained from Sigma-Aldrich Chemical Co. (Milwaukee, WI, USA). Docetaxel (DTX, purity >99 %) was purchased from Chongqing Taihao Pharmaceutical Co. Ltd (Chongqing, China). Acetonitrile (Labscan Asia, Thailand) and methanol (TEDIA Co., Inc., USA) were in HPLC grade and used without further purification. Distilled and deionized water was prepared from Millipore Water Purification System (Millipore, Milford, USA). All other reagents were of at least analytical grade.

A taxane-resistant human prostate cancer cell line (PC3-TxR cells) was provided by Department of Medicine, University of Michigan (Ann Arbor, MI, USA). It was established based on the method of Takeda *et al.* [43]. The human prostate cancer cell line (PC3 cells) was a gift from Professor Moses S. S. Chow (Center for Advanced Drug Research and Evaluation, College of Pharmacy, Western University of Health Sciences, USA). MDCK cell line transfected with human MDR1 gene (MDCK/MDR1) was a kind gift from Prof. P. Borst (The Netherlands Cancer Institute, Amsterdam, Netherlands).

### Inoculation of tumor xenografts and drug administrations

The following experimental protocol was adopted after approval by the Animal Research Ethics Committee at the Chinese University of Hong Kong. PC3 and PC3-TxR cells were cultured in RPMI 1640 medium containing 10% FBS and 1% penicillin-streptomycin in a humidified atmosphere of 5% CO_2_ at 37°C. PC3 and PC3-TxR cell pellets were re-suspended in serum free RPMI1640 and diluted by Matrigel™ Basement Membrane Matrix (BD Biosciences, Bedford, MA, USA) to the density of 3.33 × 10^6^cells/ml. Male ICR-NOD/SCID mice (Laboratory Animal Services Center at the Chinese University of Hong Kong) weighing 25 to 30 g (age 6–8 weeks) was anesthetized with an intraperitoneal injection of a cocktail containing 87 mg/kg ketamine and 8.7 mg/kg xylazine (diluted with saline and the injection volume was about 0.1 ml). The cell suspension was injected subcutaneously to both the left and right flank region. The tumors were allowed to grow for about 2 weeks. Tumor volumes were measured twice per week (tumor volume = [(W^2^ × L)/2], where W is the tumor measurement at the widest point, and L is the tumor dimension at the longest point). When the tumors reach about 100 mm^3^, treatment was started and the date was designated as Day 0.

PIP and DTX were dissolved in DMSO to yield the concentrations of 50 mg/ml and 80 mg/ml, respectively. For oral administration of PIP, in 1 ml of PIP dosing solution, 100 µl of its stock solution was solubilized with 500 µl of PEG 400 and 400 µl of physiological saline and the mixture was administrated orally to mice at the dose of 0.1 ml/10 g body weight. For intravenous administration of DTX, 50 µl of DTX stock solution was diluted with 500 µl of PEG 400, followed by 450 µl of saline, and the mixture was injected into mice via tail vein at the dose of 0.05 ml/10 g. As to the Vehicle Control of DTX and PIP, same volumes (oral 0.1 ml/10 g, intravenous 0.05 ml/10g) of physiological saline with 50% of PEG400 and 10% of DMSO were used. On Day 0, mice with PC3 cell xenografts were divided into two groups and mice with PC3-TxR cell xenografts were divided into four groups. Mice in each group were treated according to Supplementary Table 3 and the drug dosing lasted for 2 weeks with body weight and tumor sized measured twice per week. In the co-administration treatment group, PIP was orally administrated two hours before the tail vein injection of DTX. At the end of experiment, the mice were anesthetized followed by haemospasia to death by cardiac puncture.

### Quantification of drug concentration in plasma and various tissues

On Day 14, mice were sacrificed two hours post drug administration. Blood samples were collected by cardiac puncture followed by centrifugation at 5000 rpm for 3 min for obtaining the plasma. Brain, lung, liver, kidney and tumor were collected and weighed. About 100 mg of each tissue samples were carefully weighed and homogenized after being spiked with 300 µl of formic acid (0.2%, *v/v*). About 50 µl of plasma or tissue homogenates were spiked with 5 µl of 50% acetonitrile in water and 150 µl of mixture of acetonitrile and methanol (50:50, *v/v*) containing glibenclamide at 50 ng/ml as internal standard. After centrifugation at 13000 rpm for 10 min, the supernatants were injected into LC/MS/MS system which consisted of an Agilent 6430 triple quadrupole mass spectrometer equipped with an electrospray ionization source (ESI), two Agilent 1290 series pumps and autosampler (Agilent Technologies, Inc., CA, USA). The analytes were separated using a Waters Nova-Pak^Ò^C_18_ column (150 mm×3.9 mm, i.d., 4 µm particle size) protected by a collared frit (4/4.6 mm i.d., 0.5 µm pore size, Thermo Scientific full name, city, state, country). The mobile phase consisted of 0.2% formic acid and acetonitrile (50:50, *v/v*) with a flow rate at 0.4 ml/min. Data acquisition was conducted at multiple reaction monitoring (MRM), with precursor-to-product ion transitions of *m/z* 286.1→201.1 for PIP, *m/z* 830.3→548.9 for DTX and *m/z* 494.2→369.0 for glibenclamide.

### P-gp inhibition studies

P-glycoprotein (P-gp, MDR1), a well-known efflux membrane transporter relating to chemoresistance, was found overexpressed in PC3-TxR cells compared to PC3 cells (the parent sensitive cell line) [17]. Thus, the inhibitory effect on P-gp activity by PIP was examined by both MDCK/MDR1 system and DTX accumulation study in PC3-TxR cells.

MDCK cells transfected with human gene (MDCK/MDR1) were cultured in Dulbecco’s modified Eagle’s medium supplemented with 10% FBS and 1% penicillin-streptomycin. Cells were subcultured when the confluence reached 80% by trypsinization with 0.25% of trypsin-EDTA. MDCK/MDR1 cells were seeded at a density of 2 × 10^5^ cells/well to 24-well and incubated for 24 h. After pre-incubation with serial concentrations of PIP for 30 min, cells were treated with DTX at 1, 5 and 10 μM and further incubated for 5 min. After washing twice with ice-cold HBSS, cells were collected in 100 µl of formic acid (0.2%, *v/v*) using a scraper. BCA Protein Quantification Kit (Abcamplc., Cambridge, UK) was used to quantify the protein content. Cell lysate was treated by 300 µl of mixture of acetonitrile and methanol (50:50, *v/v*) containing glibenclamide at 50 ng/ml, followed by sonication for 15 min. After centrifuged at 13,000 rpm for 10 min, the supernatant was injected into LC/MS/MS system to determine the concentration of DTX.

The PC3 and PC3-TxR cells were seeded in 6-well plate with density of 1×10^4^ cells/ml and grew for 24 hours. The cells were than treated with 100 nM DTX. The cells were rinsed with cold PBS to wash off the attached DTX in cell membrane and collected at 0, 5, 15, 30, 60, 120, and 240 min. The cells were then lysed and the protein level was measured using a BCA approach (Bio Rad Laboratory, Hercules, CA, USA). The intracellular concentration of DTX was determined using an HPLC-MS/MS method. Briefly, to 0.1 ml of cell lysate, 0.3 ml of tert-butyl methyl ether was added and vortexed for 3 min. The upper organic layer was separated and evaporated to dryness. The residue was reconstituted using 0.1 ml of 80% acetonitrile and 10 μl was injected to an HPLC-MS/MS system for quantification. Paclitaxel was used as internal standard. The intracellular concentration was normalized per protein concentration. The PIP effect on intracellular concentration of docetaxel was then performed. The cells were seeded as above and incubated with 100 nM DTX in combination with 0, 5, 10, 25, and 50 μg/ml of PIP. PSC833 (10 μg/ml) was used as positive control. After 120 min, cells were collected and DTX concentration determined using the same assay method. The amount of DTX was compared among these treatment groups.

### Effect of piperine on the metabolism of DTX by mice liver microsomes: *in vitro* and *ex vivo studies*

Livers of ICR-NOD/SCID mice administrated with vehicle control or PIP were collected for the *ex vivo* metabolism study. *In vitro* metabolic reactions were performed in blank ICR mice liver. Hepatic microsomes were prepared according to the method of Hill [60]. The system of enzymatic incubation was conducted based on the condition reported by Mooiman *et al.* with minor modification [61]. Final reaction mixture (200 µl) consisted of DTX and/or PIP dissolved in DMSO (2%, *v/v*). For *ex vivo* metabolism, the concentration of DTX was employed at 2 mM. For *in vitro* metabolism, DTX at 2 μM was mixed with or without PIP (1.25 μM to 200 μM). Drug mixture was pre-incubated with mice liver microsome at 0.5 mg/ml in 50 mM phosphate buffer (pH7.4) containing 2.6 mM of NADP^+^, 7.1 mM of glucose-6-phosphate and 6.5 mM of magnesium chloride for 15 min. The reaction was initiated by the addition of glucose-6-phosphate dehydrogenase at 0.8 U/ml. After incubation for 60 min, the reaction was terminated by the addition of 200 µl of ice-cold acetonitrile/methanol (50:50, *v/v*) containing glibenclamide at 100 ng/ml. The mixture was centrifuged at 13000 rpm for 10 min and the supernatant was injected into LC/MS/MS for analysis. The formation of DTX metabolites was determined by the concentration decrease of DTX after 60 min of reaction.

### Microarray

#### RNA isolation and gene expression profiling

The microarray technique was utilized to identify the potential mechanisms related to the chemosensitizing effect of PIP in PC3-TxR cells by determining the gene expression profile at the whole genome level. PC3-TxR cells were seeded at a density of 1 × 10^5^ cells/ml in DMEM supplemented with 10% fetal bovine serum and cultured for 24 h. The cells were then treated with PIP at concentrations of 25 and 50 μg/ml (as low and high concentrations respectively) for 6 h. PIP was initially dissolved in DMSO and diluted to the target concentrations using DMEM. DMSO (0.5%) was used as the negative control (*n* = 2). Total RNA was extracted using RNeasy Mini Kit (QIAGEN, Valencia, CA, USA) according to the manufacturer’s protocol. The RNA was measured and the quality was checked using the RNA 6000 LabChip and Agilent 2100 BioAnalyzer. Only the high-quality RNA samples (e.g. RNA integrity number greater than 9.0) were used for subsequent microarray procedure performed at the Functional Genomics Core, Beckman Research Institute, City of Hope Comprehensive Cancer Center. Affymetrix Human Genome U133 Plus 2.0 arrays (Santa Clara, CA, USA) containing 54,675 probe sets detecting over 47,000 transcripts were used. The RNA samples were randomized and blinded prior to the microarray processing/analysis. The cRNA synthesis and labeling were carried out following the Affymetrix GeneChip 3′ IVT Express standard preparation protocol using 200 μg of total RNA from each sample, along with polyA spike-in controls. The cRNA samples were converted to double-stranded cDNA. After second-strand synthesis, the cDNA was purified with the GeneChip sample cleanup module (Affymetrix, Santa Clara, CA). Biotinylated cRNAs were then synthesized by *in vitro* transcription. For each sample, 10 mg of biotinylated cRNA along with hybridization spiked controls (bioB, bioC, bioD, and cre) was hybridized with Affymetrix Human Genome U133 Plus 2.0 array for 16 h at 45°C. Following hybridization, arrays were washed, stained, and then scanned with an Affymetrix GeneChipH 3000 7G scanner.

#### Microarray data processing and quality assessment

Statistical testing and additional analysis of the microarray data were conducted using Partek “Gene Expression” Workflow, i.e., Filtering, PCA, Sample Histogram, QC metrics, Gene Clustering, and ANOVA Analysis). The probe-set level expression data were summarized with Robust Multichip Average (RMA) by taking all 8 microarrays together. PCA and hierarchical clustering analysis (HCA) combined with heatmap was used to assess the quality of the microarray data by evaluating the reproducibility and variation of three replicates within each group and the differences among the three groups. The log2-transformed expression intensities of 54,675 probe sets from the 8 microarrays were used to calculate the Pearson Correlation Coefficient and Ward’s Minimum Variance method was used to calculate the distance between samples.

#### Gene ontology analysis

The differentially expressed genes (DEGs) between the treatment and control group were identified using cutoffs of *t*-test *p* value < 0.05 and fold change >2. The DEGs and probe sets were selected separately by comparing each treatment group with the control group. The dose dependent DEGs were than selected by overlapping the two DEGs sets. A functional annotation of these genes was carried out, using a gene ontology-based analysis of biological properties. The expression profiles of the DEGs (probe sets) were imported to the DAVID website, a web-based functional annotation tool (http://david.abcc.ncifcrf.gov/), to identify pathways significantly enriched with the DEGs.

### Data analyses

All data are expressed as the mean ± SE (standard error) and statistical analyses were performed using the Student’s *t*-test or One-way ANOVA.

## SUPPLEMENTARY MATERIALS FIGURES AND TABLES



## References

[1] Siegel RL, Miller KD, Jemal A (2016). Cancer statistics. CA Cancer J Clin, 2016.

[2] Chen W, Zheng R, Baade PD, Zhang S, Zeng H, Bray F, Jemal A, Yu XQ, He J (2016). Cancer statistics in China, 2015. CA Cancer J Clin.

[3] Guirgis HM (2015). The value of anticancer drugs in metastatic castrate-resistant prostate cancer: economic tools for the community oncologist. J Community Support Oncol.

[4] Nemr EG (2013). Metastatic castrate-resistant prostate cancer, dawn of a new age of management. BJU Int.

[5] Hwang C (2012). Overcoming docetaxel resistance in prostate cancer: a perspective review. Ther Adv Med Oncol.

[6] Petrylak DP, Tangen CM, Hussain MH, Lara PN, Jones JA, Taplin ME, Burch PA, Berry D, Moinpour C, Kohli M, Benson MC, Small EJ, Raghavan D (2004). Docetaxel and estramustine compared with mitoxantrone and prednisone for advanced refractory prostate cancer. N Engl J Med.

[7] Tannock IF, de Wit R, Berry WR, Horti J, Pluzanska A, Chi KN, Oudard S, Théodore C, James ND, Turesson I, Rosenthal MA, Eisenberger MA, TAX 327 Investigators (2004). Docetaxel plus prednisone or mitoxantrone plus prednisone for advanced prostate cancer. N Engl J Med.

[8] Seruga B, Ocana A, Tannock IF (2011). Drug resistance in metastatic castration-resistant prostate cancer. Nat Rev Clin Oncol.

[9] Kopczyńska E (2016). Role of microRNAs in the resistance of prostate cancer to docetaxel and pacltaxane. Contemp Oncol (Pozn).

[10] Engels FK, Sparreboom A, Mathot RA, Verweij J (2015). Potential for improvement of docetaxel-based chemotherapy: a pharmacological review. Br J Cancer.

[11] Baker SD, Sparreboom A, Verweij J (2006). Clinical pharmacokinetics of docetaxel: recent developments. Clin Pharmacokinet.

[12] Hendrikx JJ, Dubbelman AC, Rosing H, Schinkel AH, Schellens JH, Beijnen JH (2013). Quantification of docetaxel and its metabolites in human plasma by liquid chromatography/tandem mass spectrometry. Rapid Commun Mass Spectrom.

[13] Sparreboom A, Van Tellingen O, Scherrenburg EJ, Boesen JJ, Huizing MT, Nooijen WJ, Versluis C, Beijnen JH (1996). Isolation, purification and biological activity of major docetaxel metabolites from human feces. Drug Metab Dispos.

[14] Engels FK, Ten Tije AJ, Baker SD, Lee CK, Loos WJ, Vulto AG, Verweij J, Sparreboom A (2004). Effect of cytochrome P450 3A4 inhibition on the pharmacokinetics of docetaxel. Clin Pharmacol Ther.

[15] Goey AK, Meijerman I, Rosing H, Marchetti S, Mergui-Roelvink M, Keessen M, Burgers JA, Beijnen JH, Schellens JH (2014). The effect of St John’s wort on the pharmacokinetics of docetaxel. Clin Pharmacokinet.

[16] Cui J, Meng Q, Zhang X, Cui Q, Zhou W, Li S (2015). Design and synthesis of new a-Naphthoflavones as Cytochrome P450 (CYP) 1B1 inhibitors to overcome docetaxel-resistance associated with CYP1B1 overexpression. J Med Chem.

[17] Wang Z, Ravula R, Shi L, Song Y, Yeung S, Liu M, Lau B, Hao J, Wang J, Lam CW, Chow MS, Huang Y (2016). Overcoming chemoresistance in prostate cancer with Chinese medicine Tripterygium wilfordii via multiple mechanisms. Oncotarget.

[18] Liu HL, Luo R, Chen XQ, Ba YY, Zheng L, Guo WW, Wu X (2015). Identification and simultaneous quantification of five alkaloids in Piper longum L. by HPLC-ESI-MS(n) and UFLC-ESI-MS/MS and their application to Piper nigrum L. Food Chem.

[19] Huo XF, Pan H, Xu LH, Zha QB, He XH, Ouyang DY (2015). Piperine suppresses the expression of CXCL8 in lipopolysaccharide-activated SW480 and HT-29 cells via downregulating the mitogen-activated protein kinase pathways. Inflammation.

[20] Hu D, Wang Y, Chen Z, Ma Z, You Q, Zhang X, Liang Q, Tan H, Xiao C, Tang X, Gao Y (2015). The protective effect of piperine on dextran sulfate sodium induced inflammatory bowel disease and its relation with pregnane X receptor activation. J Ethnopharmacol.

[21] Sunila ES, Kuttan G (2004). Immunomodulatory and antitumor activity of Piper longum Linn. and piperine. J Ethnopharmacol.

[22] Pongkorpsakol P, Wongkrasant P, Kumpun S, Chatsudthipong V, Muanprasat C (2015). Inhibition of intestinal chloride secretion by piperine as a cellular basis for the anti-secretory effect of black peppers. Pharmacol Res.

[23] Srinivasan K (2007). Black pepper and its pungent principle-piperine: a review of diverse physiological effects. Crit Rev Food Sci Nutr.

[24] Atal CK, Dubey RK, Singh J (1985). Biochemical basis of enhanced drug bioavailability by piperine: evidence that piperine is a potent inhibitor of drug metabolism. J Pharmacol Exp Ther.

[25] Koul S, Koul JL, Taneja SC, Dhar KL, Jamwal DS, Singh K, Reen RK, Singh J (2000). Structure-activity relationship of piperine and its synthetic analogues for their inhibitory potentials of rat hepatic microsomal constitutive and inducible cytochrome P450 activities. Bioorgan Med Chem.

[26] Volak LP, Ghirmai S, Cashman JR, Court MH (2008). Curcuminoids inhibit multiple human chtochromes P45, UDP-glucuronosyltransferase, and sulfotransferase enzymes, whereas piperine is a relatively selective CYP3A4 inhibitor. Drug Metab Dispos.

[27] Singh DV, Godbole MM, Misra K (2013). A plausible explanation for enhanced bioavailability of P-gp substrates in presence of piperine: simulation for next generation of P-gp inhibitors. J Mol Model.

[28] Bhardwaj RK, Glaeser H, Becquemont L, Klotz U, Gupta SK, Fromm MF (2002). Piperine, a major constituent of black pepper, inhibits human P-glycoprotein and CYP3A4. J Pharmacol Exp Ther.

[29] Han Y, Tan T, Lim LY (2008). *In vitro* and *in vivo* evaluation of the effects of piperine on P-gp function and expression. Toxic Appl Pharmacol.

[30] Han HK (2011). The effects of black pepper on the intestinal absorption and hepatic metabolism of drugs. Expert Opin Drug Metab Toxicol.

[31] Basu S, Jana S, Patel VB, Patel H (2013). Effects of piperine, cinnamic acid and gallic acid on rosuvastatin pharmacokinetics in rats. Phytother Res.

[32] Liang YZ, Chen HM, Su ZQ, Hou SZ, Chen XY, Zheng YF, Li YC, Lin J, Zhan J, Su ZR, Fu LD (2014). White peeper and piperine have different effects on pharmacokinetics of puerarin in rats. Evid Based Complement Alternat Med.

[33] Qiang F, Kang KW, Han HK (2012). Repeated dosing of piperine induced gene expression of P-glycoprotein via stimulated pregnane-X-receptor activity and altered pharmacokinetics of diltiazem in rats. Biopharm Drug Dispos.

[34] Alhumayyd MS, Bukhari IA, Almotrefi AA (2014). Effect of piperine, a major component of black pepper, on the pharmacokinetics of domperidone in rats. J Physiol Pharmacol.

[35] Feng X, Liu Y, Wang X, Di X (2014). Effects of piperine on the intestinal permeability and pharmacokinetics of linarin in rats. Molecules.

[36] Li S, Lei Y, Jia Y, Li N, Wink M, Ma Y (2011). Piperine, a piperidine alkaloid from Piper nigrum re-sensitizes P-gp, MRP1 and BCRP dependent multidrug resistant cancer cells. Phytomedicine.

[37] Makhov P, Golovine K, Canter D, Kutikov A, Simhan J, Corlew MM, Uzzo RG, Kolenko VM (2012). Co-administration of piperine and docetaxel results in improved anti-tumor efficacy via inhibition of CYP3A4 activity. Prostate.

[38] Martinez VG, O’Connor R, Liang Y, Clynes M (2008). CYP1B1 expression is induced by docetaxel: effect on cell viability and drug resistance. Br J Cancer.

[39] Chang I, Mitsui Y, Fukuhara S, Gill A, Wong DK, Yamamura S, Shahryari V, Tabatabai ZL, Dahiya R, Shin DM, Tanaka Y (2015). Loss of miR-200c up-regulates CYP1B1 and confers docetaxel resistance in renal cell carcinoma. Oncotarget.

[40] Pastina I, Giovannetti E, Chioni A, Sissung TM, Crea F, Orlandini C, Price DK, Cianci C, Figg WD, Ricci S, Danesi R (2010). Cytochrome 450 1B1 (CYP1B1) polymorphisms associated with response to docetaxel in Castration-Resistant Prostate Cancer (CRPC) patients. BMC Cancer.

[41] Venkatesh S, Durga KD, Padmavathi Y, Reddy BM, Mullangi R (2011). Influence of piperine on ibuprofen induced antinociception and its pharmacokinetics. Arzneimittelforschung.

[42] Sama V, Nadipelli M, Yenumula P, Bommineni MR, Mullangi R (2012). Effect of piperine on antihyperglycemic activity and pharmacokinetic profile of nateglinide. Arzneimittelforschung.

[43] Takeda M, Mizokami A, Mamiya K, Li YQ, Zhang J, Keller ET, Namiki M (2007). The establishment of two paclitaxel-resistant prostate cancer cell lines and the mechanisms of paclitaxel resistance with two cell lines. Prostate.

[44] Wang Z, Ravula R, Cao M, Chow M, Huang Y (2010). Transporter-mediated multidrug resistance and its modulation by Chinese medicines and other herbal products. Curr Drug Discov Technol.

[45] Pauli-Maqnus C, Kroetz DL (2004). Functional implications of genetic polymorphisms in the multidrug resistance gene MDR1 (ABCB1). Pharm Res.

[46] Cloudhuri S, Klaassen CD (2006). Structure, function, expression, genomic organization, and single nucleotide polymorphisms of human ABCB1 (MDR1), ABCC (MRP), and ABCG2 (BCRP) efflux transporters. Int J Toxicol.

[47] Gajjar K, Martin-Hirsch P, Martin F (2012). CYP 1B1 and hormone-induced cancer. Cancer Let.

[48] McFadyen M, McLeod H, Jackson F, Melvin W, Doehmer J, Murray G (2001). Cytochrome P450 CYP1B1 protein expression: a novel mechanism of anticancer drug resistance. Biochem Pharmacol.

[49] Bournique B, Lemarié A (2002). Docetaxel (Taxotere) is not metabolized by recombinant human CYP1B1 *in vitro*, but acts as an effector of this isozyme. Drug Metab Dispos.

[50] Pradeep CR, Kuttan G (2002). Effect of piperine on the inhibition of lung metastasis induced B16F-10 melanoma cells in mice. Clin Exp Metastasis.

[51] Selvendiran K, Banu SM, Sakthisekaran D (2005). Oral supplementation of piperine leads to altered phase II enzymes and reduced DNA damage and DNA-protein cross links in Benzo(a)pyrene induced experimental lung carcinogenesis. Mol Cell Biochem.

[52] Selvendiran K, Thirunavukkarasu C, Singh JP, Padmavathi R, Sakthisekaran D (2005). Chemopreventive effect of piperine on mitochondrial TCA cycle and phase-I and glutathione-metabolizing enzymes in benzo(a)pyrene induced lung carcinogenesis in Swiss albino mice. Mol Cell Biochem.

[53] Samykutty A, Shetty AV, Dakshinamoorthy G, Bartik MM, Johnson GL, Webb B, Zheng G, Chen A, Kalyanasundaram R, Munirathinam G (2013). Piperine, a Bioactive Component of Pepper Spice Exerts Therapeutic Effects on Androgen Dependent and Androgen Independent Prostate Cancer Cells. PLoS One.

[54] Yu H, Hendrikx JJ, Rottenberg S, Schellens JH, Beijnen JH, Huitema AD (2016). Development of a tumour growth inhibition model to elucidate the effects of ritonavir on intratumoural metabolism and anti-tumour effect of docetaxel in a mouse model for hereditary breast cancer. AAPS J.

[55] Liu HL, Luo R, Chen XQ, Ba YY, Zheng L, Guo WW, Wu X (2015). Identification and simultaneous quantification of five alkaloids in Piper longum L. by HPLC-ESI-MS(n) and UFLC-ESI -MS/MS and their application to Piper nigrum L. Food Chem.

[56] Srinivasan K (2007). Black Pepper and its pungent principle-piperine: a review of diverse physiological effects. Crit Rev Food Nutri.

[57] Piyachaturawat P, Glinsukon T, Toskulkao C (1983). Acute and subacute toxicity of piperine in mice, rats and hamsters. Toxicol Lett.

[58] Lee KP, Lee K, Park WH, Kim H, Hong H (2015). Piperine inhibits platelet-derived growth factor-BB-induced proliferation and migration in vascular smooth muscle cells. J Med Food.

[59] Li G, Ruan L, Chen R, Wang R, Xie X, Zhang M, Chen L, Yan Q, Reed M, Chen J, Xu Y, Pan J, Huan W (2015). Synergistic antidepressant-like effect of ferulic acid in combination with piperine: involvement of monoaminergic system. Metab Brain Dis.

[60] Hill JR *In vitro* drug metabolism using liver microsomes. Curr Protoc Pharmacol.

[61] Mooiman KD, Maas-Bakker RF, Hendrikx JJ, Bank PC, Rosing H, Beijnen JH, Schellens JH, Meijerman I (2014). The effect of complementary and alternative medicines on CYP3A4-mediated metabolism of three different substrates: 7-benzyloxy-4-trifluoromethyl-coumarin, midazolam and docetaxel. J Pharm Pharmacol.

