# Supplementary material for: Enhanced anti-tumor efficacy and mechanisms associated with docetaxel-piperine combination- in vitro and in vivo investigation using a taxane-resistant prostate cancer model
Source: Oncotarget. 2017 Dec 14;9(3):3338–52. doi: 10.18632/oncotarget.23235 (PMC5790467; doi:10.18632/oncotarget.23235)
Supplement: Supplementary file 1 [file oncotarget-09-3338-s001.pdf]

Enhanced anti-tumor efficacy and mechanisms associated with docetaxel-piperine combination- *in vitro* and *in vivo* investigation using a taxane-resistant prostate cancer model

SUPPLEMENTARY MATERIALS

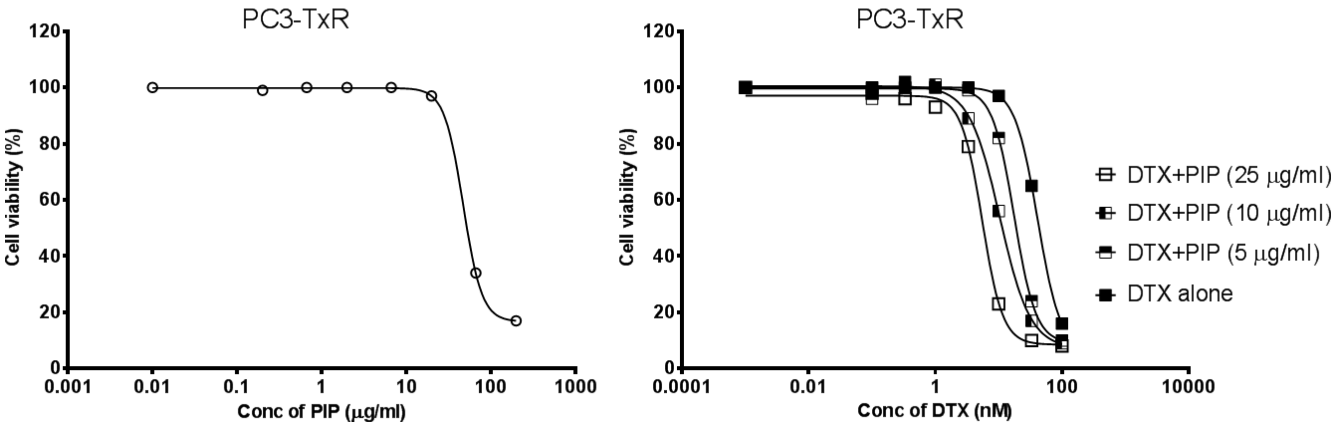

Supplementary Figure 1: Cell viability (measured from triplicate samples) of PC3-TxR cells. (**A**) The cytotoxicity of PIP on PC3-TxR (IC50 47.3 μg/ml); (**B**) The cytotoxicity of DTX on PC3-TxR alone or in combination with PIP at concentrations of 5, 10, and 25 μg/ml (IC50 values of DTX are 41.4, 17.6, 10.4, and 5.5 nM for DTX alone, DTX + 5 μg/ml PIP, DTX + 10 μg/ml PIP, and DTX + 25 μg/ml PIP, respectively). Abbreviation: DTX = docetaxel; PIP = piperine.

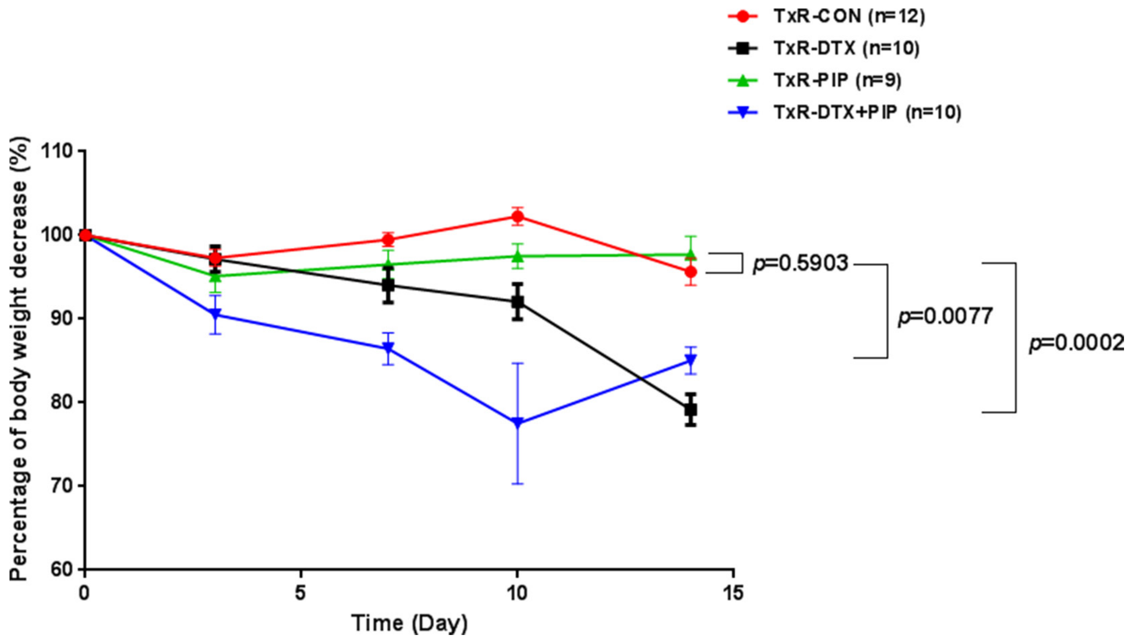

Supplementary Figure 2: Plot of percentage of body weight decrease (%) versus time (Day) in mice with TxR tumor xenografts after treatment with saline (*n* = 12), DTX (20 mg/kg, *n* = 10), PIP (50 mg/kg, *n* = 9), or co-administration of DTX and PIP (20 mg/kg DTX and 50 mg/kg PIP, *n* = 10). Abbreviation: DTX = docetaxel; PIP = piperine.

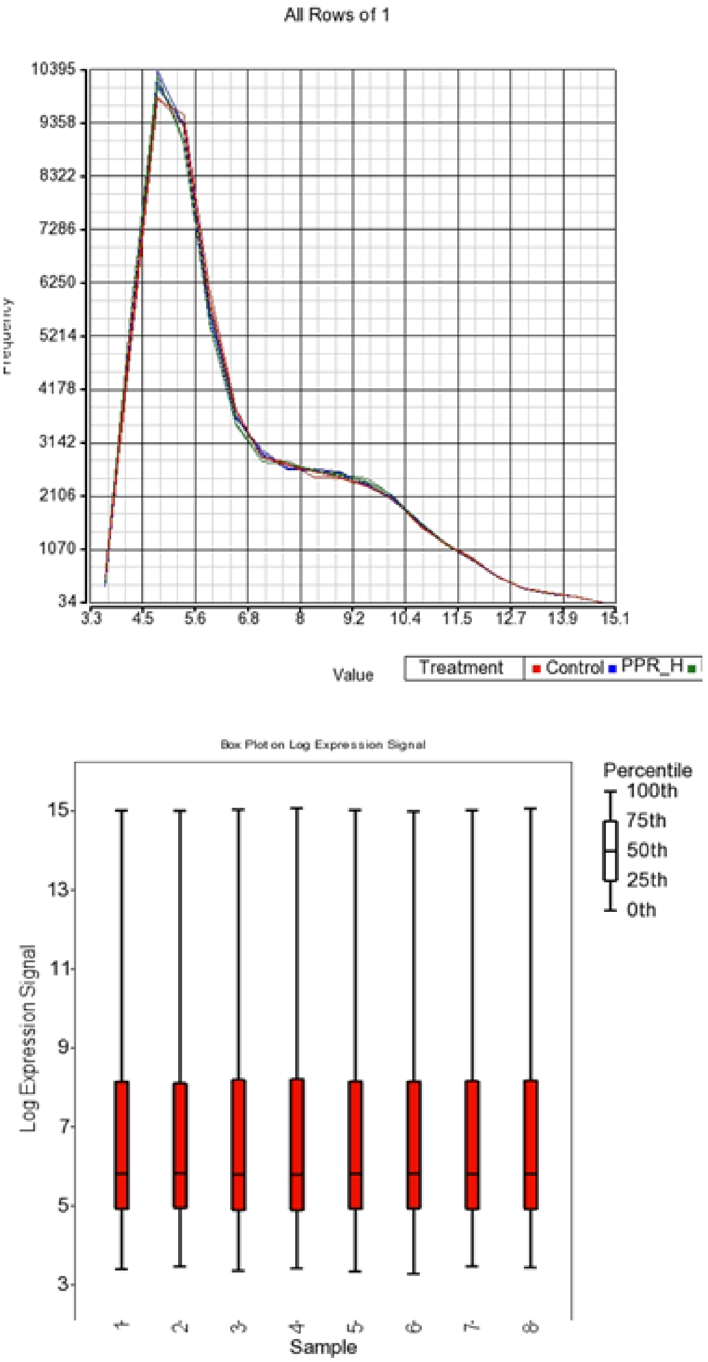

Supplementary Figure 3: Sample histograms (upper) and intensity distribution (bottom) of microarray data.

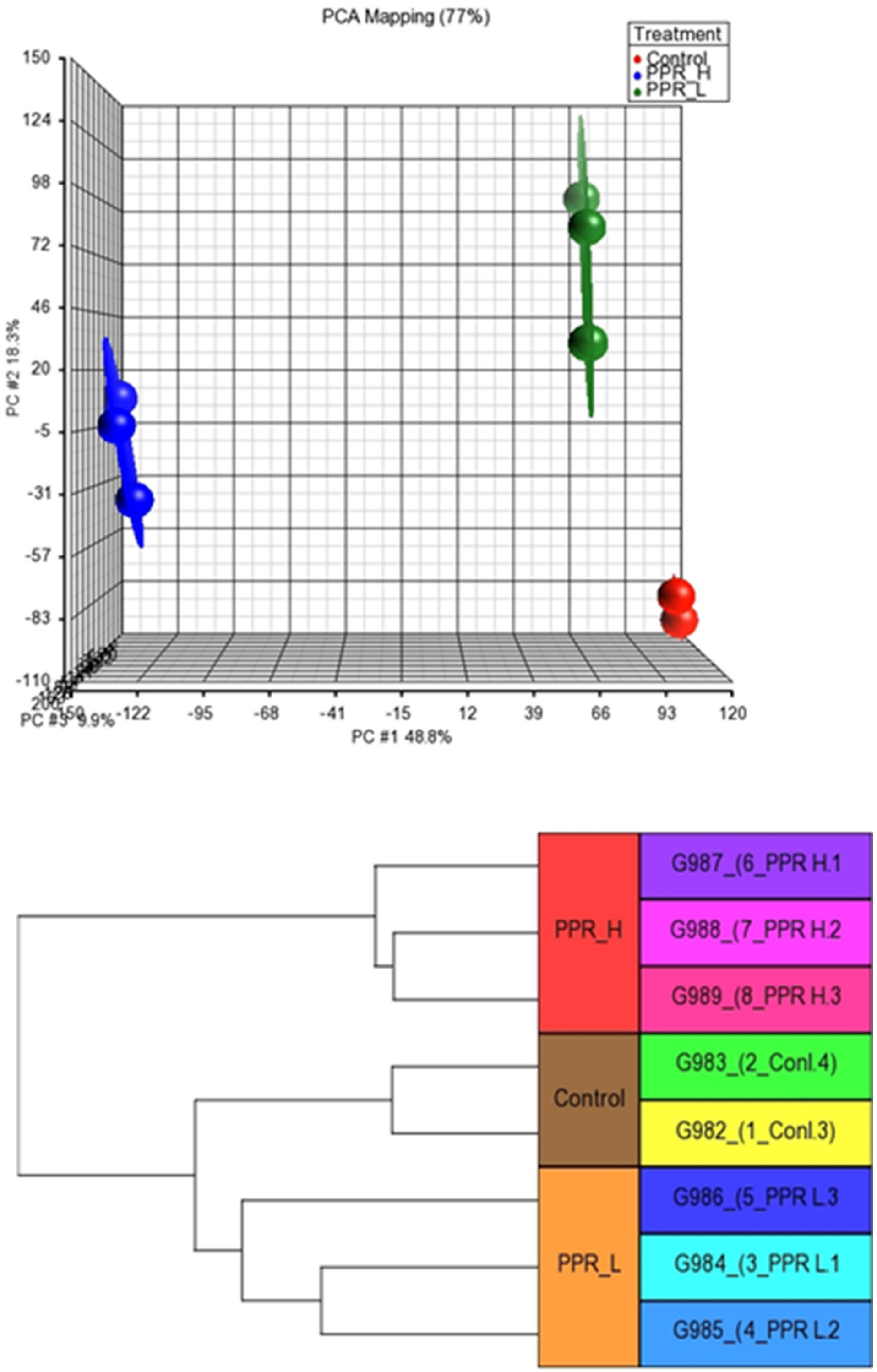

Supplementary Figure 4: PCA and hierarchical cluster analysis.

Supplementary Table 1: IPA results for piperine high concentration treatment vs. control

**Top canonical pathways**				
**Regulation**	**Name**	***p*-value**	**Overlap**	
**Up**	Unfolded protein response	8.27E-06	13.2%	(7/53)
	Adipogenesis pathway	5.24E-05	7.4%	(9/122)
	Cysteine Biosynthesis/Homocysteine Degradation	1.93E-04	100.0%	(2/2)
	Role of Macrophages, Fibroblasts and Endothelial Cells in Rheumatoid Arthritis	2.05E-03	3.9%	(11/282)
	VDR/RXR Activation	4.39E-03	6.5%	(5/77)
**Down**	14-3-3-mediated Signaling	1.12E-04	5.2%	(8/154)
	Germ Cell-Sertoli Cell Junction Signaling	2.13E-04	4.7%	(8/169)
	Sertoli Cell-Setoli Cell Junction Signaling	3.42E-04	6.1%	(6/98)
	p53 Signaling	3.52E-04	7.8%	(5/64)
	Remodeling of Epithelial Adherens Junctions			

**Top upstream regulators**			
**Regulation**	**Upstream eegulator**	***p*-value of overlap**	**Predicted activation**
**Up**	tosedostat	7.18E-19	Activated
	ATF4	2.55E-13	Activated
	TRIB3	1.67E-11	Inhibited
	miR-30c-5p (and other miRNAs w/seed GUAAACA)	4.12E-11	Inhibited
	thapsigargin	5.20E-11	Activated
**Down**	miR-23a-3p (and other miRNAs w/seed UCACAUU)	1.91E-11	Activated
	miR-124-3p (and other miRNAs w/seed AAGGCAC)	1.08E-10	Activated
	miR-19b-3p (and other miRNAs w/seed GUGCAAA)	2.94E-08	Activated
	miR-200b-3p (and other miRNAs w/seed AAUACUG)	1.33E-07	Activated
	miR-26a-5p (and other miRNAs w/seed UCAAGUA)	2.01E-07	Activated

Supplementary Table 2: IPA results for piperine low concentration treatment vs. Control

Top canonical pathways				
**Regulation**	**Name**	***p*-value**	**Overlap**	
**Up**	HIF1 Signaling	2.45E-03	3.00%	(3/100)
	Tight Junction Signaling	9.69E-03	1.80%	(3/164)
	Hutington's Disease Signaling	2.25E-02	1.30%	(3/225)
	Guanosine Nucleotides Degradation III	3.44E-02	7.70%	(1/13)
	Urate Biosynthesis/Inosine 5′-phosphate Degradation	3.70E-02	7.10%	(1/14)
**Down**	Bupropion Degradation	7.28E-04	8.30%	(2/24)
	Acetone Degradation I (to Methylglyoxal)	7.90E-04	8.00%	(2/25)
	HMGB1 Signaling	9.84E-04	2.50%	(3/118)
	Atherosclerosis Signaling	9.84E-04	2.50%	(3/118)
	Estrogen Biosynthesis	1.55E-03	5.70%	(2/35)

Top upstream regulators			
**Regulation**	**Upstream regulator**	***p*-value of overlap**	**Predicted activation**
**Up**	PPARG	1.58E-08	Activated
	troglitazone	2.02E-06	Activated
	fluticasone	4.15E-06	
	PLAGL1	6.72E-06	
	Insulin	6.74E-06	
**Down**	CD40	1.84E-10	
	5-hydroxytryptamine	4.10E-10	Inhibited
	TREM1	5.36E-10	
	TNF	6.54E-10	
	U0126	9.03E-10	

Supplementary Table 3: Drug administrations for each treatment group with different cell xenografts

Treatment Groups	Cell xenografts	Drug administration
PC3-CON	PC3	Vehicle, Daily, p.o.
PC3-DTX	PC3	DTX, 20 mg/kg, once a week, i.v.
TxR-CON	PC3-TxR	Vehicle, Daily, p.o.
TxR-DTX	PC3-TxR	DTX, 20 mg/kg, once a week, i.v.
TxR-PIP	PC3-TxR	PIP, 50 mg/kg, daily, p.o.
TxR-DTX+PIP	PC3-TxR	DTX at 20 mg/kg (once a week, i.v.) and PIP at 50 mg/kg (daily, p.o.)

Abbreviation: DTX = docetaxel; PIP = piperine.
